# Nonlinear Coupling between Cortical Oscillations and Muscle Activity during Isotonic Wrist Flexion

**DOI:** 10.3389/fncom.2016.00126

**Published:** 2016-12-06

**Authors:** Yuan Yang, Teodoro Solis-Escalante, Mark van de Ruit, Frans C. T. van der Helm, Alfred C. Schouten

**Affiliations:** ^1^Neuromuscular Control Laboratory, Department of Biomechanical Engineering, Delft University of TechnologyDelft, Netherlands; ^2^MIRA Institute for Biomedical Technology and Technical Medicine, University of TwenteEnschede, Netherlands

**Keywords:** corticomuscular coupling, nonlinear coherence, sensorimotor system, EEG, EMG

## Abstract

Coupling between cortical oscillations and muscle activity facilitates neuronal communication during motor control. The linear part of this coupling, known as corticomuscular coherence, has received substantial attention, even though neuronal communication underlying motor control has been demonstrated to be highly nonlinear. A full assessment of corticomuscular coupling, including the nonlinear part, is essential to understand the neuronal communication within the sensorimotor system. In this study, we applied the recently developed n:m coherence method to assess nonlinear corticomuscular coupling during isotonic wrist flexion. The n:m coherence is a generalized metric for quantifying nonlinear cross-frequency coupling as well as linear iso-frequency coupling. By using independent component analysis (ICA) and equivalent current dipole source localization, we identify four sensorimotor related brain areas based on the locations of the dipoles, i.e., the contralateral primary sensorimotor areas, supplementary motor area (SMA), prefrontal area (PFA) and posterior parietal cortex (PPC). For all these areas, linear coupling between electroencephalogram (EEG) and electromyogram (EMG) is present with peaks in the beta band (15–35 Hz), while nonlinear coupling is detected with both integer (1:2, 1:3, 1:4) and non-integer (2:3) harmonics. Significant differences between brain areas is shown in linear coupling with stronger coherence for the primary sensorimotor areas and motor association cortices (SMA, PFA) compared to the sensory association area (PPC); but not for the nonlinear coupling. Moreover, the detected nonlinear coupling is similar to previously reported nonlinear coupling of cortical activity to somatosensory stimuli. We suggest that the descending motor pathways mainly contribute to linear corticomuscular coupling, while nonlinear coupling likely originates from sensory feedback.

## Introduction

Coupling between neuronal populations facilitates their communication in the nervous system and may shorten reaction times (Varela et al., 2001; Schoffelen et al., 2005). When performing a simple muscle contraction, the human sensorimotor cortices typically generate oscillatory activity coupled with muscle activity (Conway et al., 1995; Kristeva et al., 2007). Corticomuscular coupling plays an important role for neuronal communication between central and peripheral sensorimotor systems (Salenius and Hari, 2003; van Wijk et al., 2012). This is highlighted in many pathological cases where abnormal corticomuscular coupling indicates impaired neuronal communication leading to both motor and sensory impairments (Caviness et al., 2003; Grosse et al., 2003; Brown, 2007; Fang et al., 2009). Thus, a full assessment of corticomuscular coupling is essential to understand neuronal communication within the sensorimotor system, and could contribute to clinical studies related to motor disorders and rehabilitation.

The linear part of corticomuscular coupling, known as corticomuscular coherence, has received much attention for decades (Mima and Hallett, 1999; Schoffelen et al., 2005; Witham et al., 2011; Raethjen and Muthuraman, 2012), although neuronal communication underlying motor control have been demonstrated to be highly nonlinear (Darvas et al., 2009; Chen et al., 2010; Vlaar et al., 2016; Yang et al., 2016c). Several studies suggest that nonlinear coupling plays an equally important role as linear coupling in neuronal communication (Friston, 2001; Breakspear et al., 2003; Chen et al., 2010). Moreover, the clinical relevance of nonlinear coupling has also been demonstrated (Sanger et al., 2002; He et al., 2016). In this work, we use a recently developed method, i.e., n:m coherence (Yang et al., 2015, 2016a), to assess nonlinear, as well as linear, corticomuscular coupling during an isotonic wrist flexion. The n:m coherence is a straightforward extension of the linear coherence used in the corticomuscular coherence (Mima and Hallett, 1999) based on high-order statistics (Nikias and Mendel, 1993) for investigating both nonlinear and linear correlation between signals. Thus, the linear part of our results obtained by this method would be comparable to previous corticomuscular coherence studies. Using an established experimental paradigm completed by healthy subjects, our motivation is to establish a reference for future clinical studies.

In general, motor actions can be either voluntary or reflexive. Recently, nonlinear neuronal synchronization has been demonstrated in the human stretch reflex induced by mechanical perturbation (Yang et al., 2016c). In this study, we focus on voluntary motor control. In contrast to reflexive movement, voluntary motor actions start with intent rather than sensory feedback. The descending corticospinal motor tract is an obvious pathway resulting in the corticomuscular coupling. Furthermore, neural oscillations coupled with muscle activity are found in the primary motor cortex as well as motor association cortices including the supplementary motor area (SMA) and the prefrontal cortex (Ohara et al., 2001; Babiloni et al., 2008; Meng et al., 2008; Chen et al., 2013).

During a motor task, the output from the neuromuscular system (e.g., force, velocity of movement) is sensed by the mechanoreceptors in the periphery (i.e., muscle spindles and Golgi tendon organs) to provide somatosensory feedback (Scott, 2004). This somatosensory feedback can lead to an ascending conduction of neuronal oscillations from the periphery to the cortex (Baker et al., 2006; Witham and Baker, 2007; Witham et al., 2007, 2011). Thereby, coupled oscillations have also been detected in the somatosensory cortex and sensory association areas such as posterior parietal cortex (PPC; Witham et al., 2007, 2010; Meng et al., 2008). Thus, corticomuscular coupling is mediated in a closed-loop (Schouten and Campfens, 2012; van Wijk et al., 2012; Campfens et al., 2014).

Many studies have demonstrated nonlinear cross-frequency coupling between cortical oscillations and external somatosensory input (Snyder, 1992; Tobimatsu et al., 1999; Yang et al., 2016b). Specially, both integer (n*f_i_) and non-integer (n*f_i_/m, also known as n:m coupling) harmonic coupling has been reported in the EEG when healthy subjects receive periodic somatosensory input with a certain frequency f_i_ (Langdon et al., 2011). These findings indicate a nonlinear neuronal mechanism in ascending sensory tracts in presence of external stimuli.

We hypothesize that corticomuscular coupling could be mediated by the nonlinear neuronal mechanism of ascending sensory tracts, even without external stimuli, and therefore shows nonlinear behavior in the closed-loop. To investigate this hypothesis, we recorded electromyogram (EMG) and high-density electroencephalogram (EEG) from 11 healthy volunteers during isotonic flexion of the right wrist. Without using any external somatosensory stimulus, our study aims to reveal the intrinsic neuronal interaction between the cerebral cortex and motor units of the forearm during the voluntary motor task, expressed as corticomuscular coupling. Performing independent component analysis (ICA) and equivalent current dipole source localization, we assessed corticomuscular coupling within the sensorimotor network involving the primary sensorimotor areas and sensorimotor association areas.

## Materials and Methods

### Subjects

Eleven healthy volunteers (24 ± 3 years of age, 4 female) participated in the experiment. All subjects were right-handed. Written informed consent was provided by all subjects. All procedures were approved by the Human Research Ethics Committee of the Delft University of Technology and are in accordance with the Helsinki Declaration of 1975, as revised in 2008 (Williams, 2008).

### Experimental Protocol

**Figure 1:**
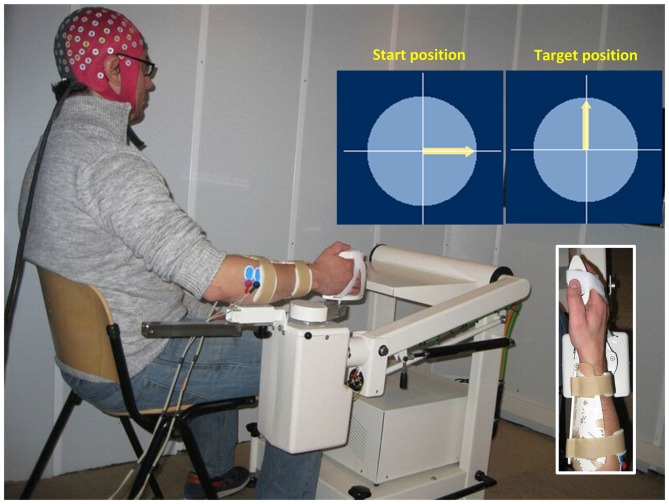
**Illustration of the experimental setup.** The subject’s right hand is attached to the handle of a wrist manipulator and the lower arm is firmly fixed in place. When the trial starts, an arrow appears in the center of screen to provide visual feedback on the exerted torque. The subject is instructed to keep the arrow pointing upwards (1 Nm flexion torque). EEG and EMG are recorded simultaneously during the task.

The experiment was performed inside a dim sound-proof cabin (Esmono Sound B.V., Dongen, Netherlands). Figure 1 shows the experimental setup. Subjects sat comfortably next to a wrist manipulator (Wristalyzer, Moog Inc, Nieuw-Vennep, Netherlands), which contains a force transducer for measuring wrist torque. The forearm of the subject was strapped to the arm rest, while the right hand rested on the handle of manipulator (fixed with straps to remove the need for grasping). Subjects were instructed to perform an isotonic flexion (1 Nm) using their right wrist. The level of muscle contraction was 10%–15% of the maximum voluntary flexion torque of each subject. The experiment included 60 trials, lasting 22 s each. When the trial started, an arrow appeared on screen to provide visual feedback on the exerted torque. The angle of the arrow was proportional to the torque exerted by the subjects, with 1 Nm corresponding to 90° (arrow pointing upwards). Between trials, there was a random pause from 10 s to 12 s, where the subjects were instructed to relax their arm and hand.

### Data Acquisition

High-density EEG was measured using a 128-channel cap (5/10 systems, WaveGuard cap, ANT Neuro, Netherlands) with Al/AgCl electrodes. EMG signals were measured from the extensor and flexor carpi radialis muscles of the right forearm using bipolar derivations, i.e., two Ag/AgCl electrodes placed on the muscle belly with 2 cm inter-electrode distance. EEG, EMG and wrist torque were recorded simultaneously at 2048 Hz using a bio-signal amplifier (Refa System, TMSi, Netherlands). The amplifier contains an antialiasing low-pass filter with cut-off frequency of 552 Hz.

### Data Analysis

#### Preprocessing

The EEG and EMG were first filtered by a band-pass (1–200 Hz) zero-phase shift FIR filter using EEGLAB (version: 13.2.2b; Delorme and Makeig, 2004) and then downsampled to 512 Hz. We segmented the data from each trial into consecutive epochs of 1 s. We rejected the first and the last epochs in each trial, as well as epochs with the variance of wrist torque larger than 1% of required flexion torque, leaving at least 860 epochs (1035 epochs on average) for each subject for further analysis.

#### Independent Component Analysis on EEG

A few EEG channels near the neck were removed due to excessive muscle artifacts. Afterwards, the EEG data were re-referenced to the common average of the remaining channels. We performed blind source separation analysis on the EEG data using the Informax ICA from EEGLAB (Delorme and Makeig, 2004). ICA algorithms have proven capable of separating artifact components from EEG signals (Jung et al., 2000) while disentangling biologically plausible cortical sources (Delorme et al., 2012). We used Informax ICA, which is widely used in neuroscience studies and proved to be a reliable algorithm (Delorme and Makeig, 2004). We computed the power spectrum of each independent component (IC) and excluded the ICs with abnormal power spectra. Typical EEG power spectra show 1/f characteristics and some spectra may have an alpha band peak around 10 Hz. High power at higher frequencies (above 20 Hz) or sharp peaks in the spectrum indicates an artifact component (Jung et al., 1998; Delorme et al., 2007). ICs with typical eye-region scalp maps were also removed (Delorme and Makeig, 2004). We removed artifact components to improve the signal-to-noise of EEG, which does not result in negative effects on later analysis including coherence and statistical analysis. The biologically plausible ICs typically have their scalp maps nearly fitting the projection of a single equivalent current dipole, and therefore their sources should be able to be localized by the dipole fitting algorithm (Delorme et al., 2012). Based on this knowledge, we used the DIPFIT algorithm (Oostenveld et al., 2010) to estimate a best-fitting single equivalent current dipole for the scalp map of each remaining IC. A standardized three-shell (i.e., skin, skull and cortex) boundary element head model extracted from the Montreal Neurological Institute (MNI) canonical template brain was used. Standard electrode locations corresponding to 5/10 system were aligned with this head model. We rejected ICs with an associated dipole located outside the brain or with residual variance larger than 10%. We used Talairach Client toolbox^1^ to identify the nearby cortex for each dipole. Afterwards, we visually examined all components to further reject the components with the dipoles located in the deep brain areas, since previous studies indicated the difficulty of localizing a deep source from the scalp EEG (Yao and Dewald, 2005; Bradley et al., 2016).

We grouped the rest of ICs across subjects based on scalp maps, estimated dipoles and power spectra (1–200 Hz) of ICs using principal component analysis and the *k*-means clustering, in order to identify the similar ICs for different subjects for group analysis. The *k* value (*k* = 10) was set to have an IC cluster with the dipole sources located around the primary sensorimotor areas for all subjects, which is in line with previous findings that all subjects have active cortical sources in the primary sensorimotor areas during a voluntary motor control task (Witham et al., 2011). ICs were identified as outliers if their locations in the clustering vector space were larger than five times of standard deviation from the obtained cluster centers. Only clusters including ICs from more than half of the subjects (i.e., at least six subjects) were used for further analysis (Wagner et al., 2016).

#### EMG Rectification

Since participants were required to produce a flexion torque, we analyzed the EMG recorded from flexor carpi radialis muscle. There is an ongoing debate on EMG rectification for computing (linear) corticomuscular coherence. Rectification of EMG is thought to improve the detection of beta-band corticomuscular coherence (Halliday et al., 1995; Myers et al., 2003; Farina et al., 2013), while several studies argued that rectification is a nonlinear process that distort the EMG spectrum (Neto and Christou, 2010; McClelland et al., 2012). Nevertheless, recent studies demonstrated that there was no difference in coherence estimates between rectified and non-rectified EMG (Yao et al., 2007; Bayraktaroglu et al., 2011). Notably, all these studies focused on the linear corticomuscular coherence. Here we computed corticomuscular coupling with both non-rectified and rectified EMG for a comparison, as there are no references for the effect of EMG rectification on nonlinear corticomuscular coupling. For rectified EMG, we applied zero-phase shift high-pass (cut-off frequency: 5 Hz) and notch (50 Hz) filters to remove possible movement and power-line artifacts in the EMG before full-wave rectification.

#### n:m Coherence Analysis

The n:m coherence is a generalized coherence measure for quantifying cross-frequency coupling between two frequency components (Yang et al., 2015, 2016a). Set *X* be the Fourier Transform of one IC of EEG, *Y* be EMG (non-rectified or rectified), n:m coherence (*nmC*) between them is computed as

(1) $$nmC(f_{X},f_{Y})\;=\;\frac{\left|S_{XY}(f_{X},f_{Y})\right|}{\sqrt{S_{XX}^{n}(f_{X})S_{YY}^{m}(f_{Y})}}$$

where *f_X_, f_Y_* in the range of 1–200 Hz, n:m = *f_Y_*: *f_X_*. $S_{XX}^{n}$(*f_X_*) is the *n*-th order auto-spectra:

(2) $$\begin{array}{l} S_{XX}^{n}(f_{X})=\;<X^{n}(f_{X}){\left(X^{n}(f_{X})\right)}^{*}>\;\\ \;=\;<{\left|\underset{n}{\underset{︸}{X(f_{X})•X(f_{X})•\cdot \cdot \cdot •X(f\_X)}}\right|}^{2}> \end{array}$$

where <·> represents the averaging over segments, and *S_XY_* (*f_X_, f_Y_*) is the n:m cross-spectrum:

(3) $$S_{XY}(f_{X},f_{Y})=<X^{n}(f_{X}){\left(Y^{m}(f_{Y})\right)}^{*}>$$

The n:m coherence reflects the strength of nonlinear cross-frequency coupling between signals. According to Cauchy-Schwarz-inequality, we have:

(4) $$\begin{array}{l} \left|<X^{n}(f_{X}){\left(Y^{m}(f_{Y})\right)}^{*}>\right|\leq \\ \;{\left(<{\left|X^{n}(f_{X})\right|}^{2}>\right)}^{1/2}{\left(<{\left|Y^{m}(f_{Y})\right|}^{2}>\right)}^{1/2} \end{array}$$

Thus, n:m coherence is bounded by 0 and 1, where 1 indicates that two signals are perfectly coupled for the given frequency pair (*f*_X_, *f*_Y_). More details on the calculations of n:m coherence can be found in a previous study (Yang et al., 2016a). The n:m coherence values in the frequency pairs involving power-line frequency (50 Hz) and its harmonics (100, 150 and 200 Hz) are set to zeros to eliminate the contamination of power-line artifact on n:m coherence.

#### Statistical Analysis

Significance of the n:m coherence values was determined by a permutation test (Bayraktaroglu et al., 2011), where we randomly shuffled EMG segments with respect to EEG segments. This approach is better than using a threshold obtained from two independent white noise signals, whose spectra are different from EEG/EMG signals. We performed 1000 permutations to get a distribution of n:m coherence for each frequency pair (*f*_X_, *f*_Y_). The estimated n:m coherence from non-permutated data is considered to be significant if its value exceeds the 95% confidence interval of the n:m coherence computed from permutated data.

We used SPSS (Version 22, IBM) to perform the statistical analysis regarding to result comparison. We performed the paired sample *t*-test to check statistically significant differences between using non-rectified and rectified EMG to estimate the cross-frequency coupling between EEG and EMG per frequency pair. Bonferroni correction was applied to control the type I error in the paired sample *t*-test (family-wise error rate *P* < 0.05). Statistically significant differences among IC clusters were examined by a one-way analysis of variance (ANOVA) with the factor “cluster” (significant level *P* < 0.05). Due to unequal sizes of samples among IC clusters, the Brown-Forsythe test of the equality of means was employed when the homogeneity of variances was violated. Additionally, the ANOVA with repeated measures was performed to check the statistical significance of nonlinearity (details about factors and levels were provided in the “Results” Section). The Greenhouse-Geisser correction was made when the sphericity was violated in the repeated measures.

## Results

**Figure 2:**
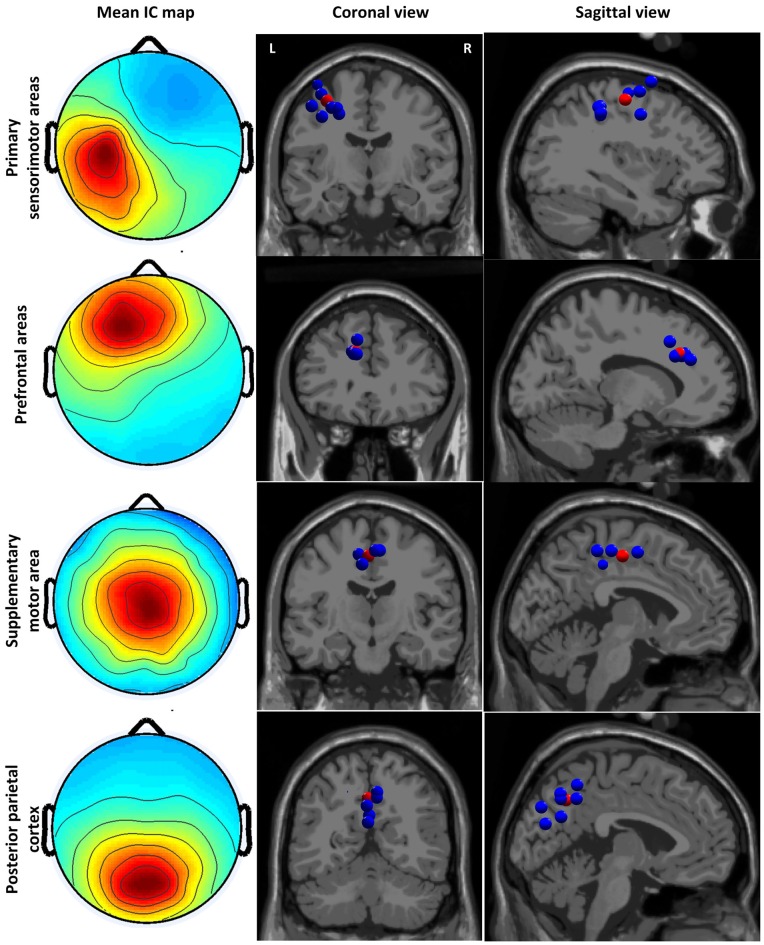
**Cluster-mean scalp projections and the location of equivalent dipole sources for different clusters.** The first column shows the cluster-mean scalp projections. The second and third columns present the location of equivalent dipole sources for each cluster in the coronal and sagittal slides, respectively. The red point indicates the mean dipole location. Coronal and sagittal slides are given in correspondence of the mean dipole position.

We identified four clusters containing components from at least six subjects with the dipoles located in the sensory and motor related cortices (see Figure 2). The IC cluster in the left primary sensorimotor areas (S1-M1) contains components from 11 subjects (one component from each subject, the same for below) with the mean dipole located in Brodmann area (BA) 4: [−33, −14, 47] (Talairach coordinates, unit: mm). The IC cluster in left prefrontal area (PFA) contains components from 6 subjects with the mean dipole located nearby BA 9: [−13, 29, 25]. The IC cluster in the SMA contains components from seven subjects with the mean dipole located in BA 6: [−3, −14, 46]. The IC cluster in the PPC contains components from nine subjects with the mean dipole located nearby BA 7: [−1, −57, 39].

**Figure 3:**
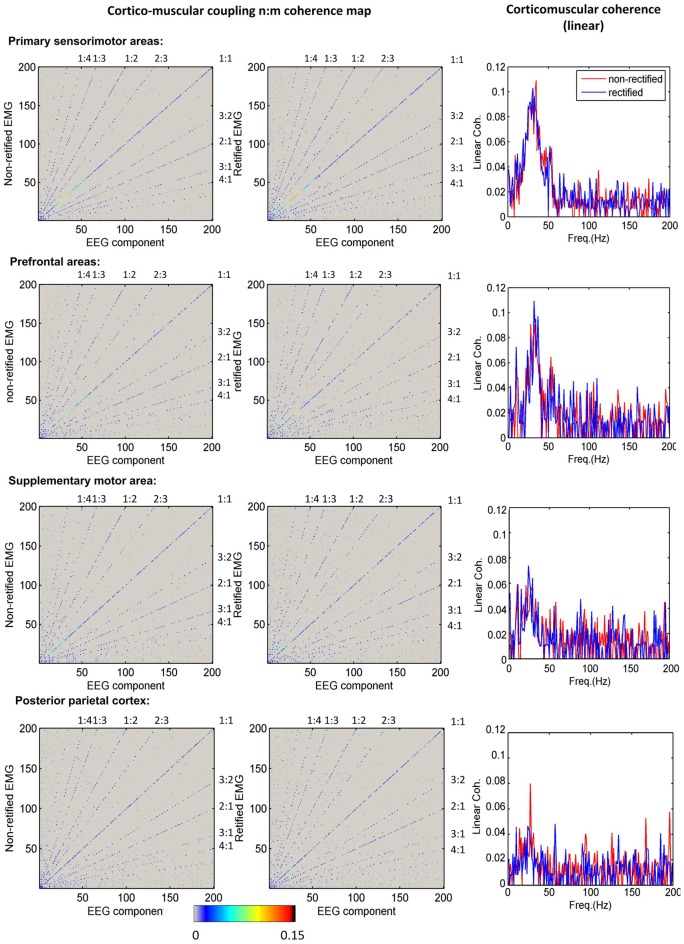
**Corticomuscular coupling for different brain areas.** The first column shows the mean maps of significant n:m coherence between independent component analysis (ICA) components and non-rectified EMG for each cluster. The second column indicates the mean maps of significant n:m coherence between ICA components and rectified EMG for each cluster. The diagonal shows the linear corticomuscular coherence. The nonlinear coupling ratio is given as EEG frequency over EMG frequency. The third column presents the comparison of linear corticomuscular coherence using non-rectified and rectified EMG.

Figure 3 shows the grand average of significant n:m coherence (across subjects within a cluster) between ICs and non-rectified/rectified EMG for each cluster. No significant differences between non-rectified and rectified EMG were found (*P* > 0.05 for family-wise error rate). The diagonal (1:1) indicates linear coupling, the rest in the map shows nonlinear coupling. To improve visualization, we also plotted linear coupling as a frequency spectrum in the third column of Figure 3. Linear coupling between EEG and EMG is present with peak values in the beta band for all sensorimotor related cortical areas. Nonlinear cross-frequency coupling between EEG and EMG is mainly shown in frequency ratios (f_EEG_:f_EEG_) of 1:2, 1:3, 1:4, 2:3, 3:2, 2:1, 3:1 and 4:1, for both non-rectified and rectified EMG in all clusters.

**Figure 4:**
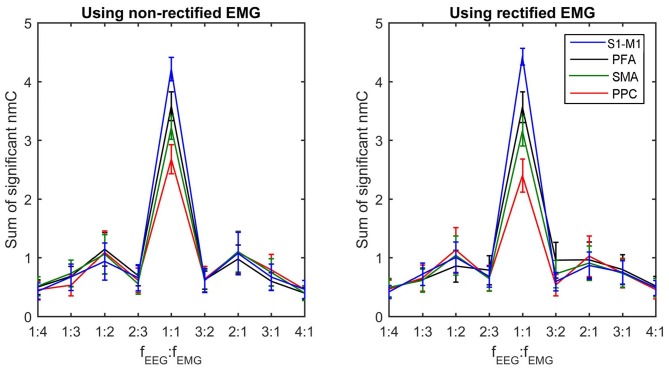
**Sum of significant n:m coherence values for each coupling ratio at each brain area.** The sum of significant coherence values indicates the overall coupling strength for each coupling ratio. The grand averages of these values across subjects within each cluster and their standard deviations (error bars) are shown.

To compare the difference of corticomuscular coupling at different coupling ratios and different brain areas, we computed the sum of all significant n:m coherence values at the same coupling ratio for each brain areas. Noteworthy, the sum value is determined not only by the strength of coupling at each individual frequency pair but also by the number of frequency pairs at the corresponding nonlinear ratio that have significant n:m coherence values. Thus, the sum value reflects the overall coupling strength for each ratio. Figure 4 shows the grand average of this sum value (across subjects within a cluster) for each coupling ratio and each brain area. Using one-way ANOVA with the factor “cluster” (four clusters), significant differences among clusters are detected in the linear coupling for both non-rectified (*F*_(3,19.545)_ = 4.133, *P* = 0.020) and rectified EMG (*F*_(3,18.054)_ = 4.259, *P* = 0.019), showing the highest sum value for the cluster in the S1-M1 and the lowest value for the cluster in the PPC. Removing the beta-band (15–35 Hz) linear coherence, this effect becomes insignificant, indicating that the significance is mainly related to beta-band coupling. No significant differences among clusters are found for nonlinear coupling.

A two-way ANOVA with factors “ratios” (nine frequency ratios) and “rectification” (non-rectification vs. rectification) was performed to check the difference of the sum of n:m coherence values among difference ratios across subjects and ICs. The effect of ratio on the sum value is significant (*F*_(8,256)_ = 74.625, *P* < 0.001), while the effect of rectification is insignificant. To check the difference between a ratio *n*:*m* and its inverse *m*:*n*, a three-way ANOVA with factor “nonlinear order” (four orders, i.e., 2nd, 3rd, 4th and a non-integer order), “asymmetry” (*n*:*m* vs. *m*:*n*) and “rectification” (non-rectification vs. rectification) was also performed for the nonlinear coupling alone. Only the effect of nonlinear order is statistically significant (*F*_(2.772,88.698)_ = 141.064, *P* < 0.001).

## Discussion

In this study, we used a recently developed cross-frequency coupling method, n:m coherence, to reveal nonlinear corticomuscular coupling for the first time. The n:m coherence is a generalized coherence measure incorporating both phase and amplitude relationship (Yang et al., 2015, 2016a). A recent study found that EEG oscillations, originating from the primary sensorimotor areas, can transmit not only the phase but also amplitude dynamics through the spinal motoneurons down to the periphery (Bayraktaroglu et al., 2013). Thus, the n:m coherence is more suitable to assess the corticomuscular coupling than other cross-frequency coupling measures purely assessing phase or amplitude relationships (Young and Eggermont, 2009). Noteworthy, the n:m coherence is also different from *nested oscillation* measures, which reflect that the phase of a slower oscillation modulates the amplitude of a faster oscillation and are more often used for cognitive studies, such as learning and memory (see Aru et al., 2015 for more details about nested oscillation measures). Using the n:m coherence, our work provides a full assessment of corticomuscular coupling including both linear and nonlinear parts in the sensorimotor network involving the primary sensorimotor cortices and association areas.

### Linear Corticomuscular Coupling

In line with previous studies (Babiloni et al., 2008; Meng et al., 2008; Witham et al., 2010; Chen et al., 2013), beta-band corticomuscular coherence (linear) is detected not only at the primary sensorimotor areas (S1-M1) but also association areas, including both motor association cortices, i.e., PFA and SMA and sensory association area, i.e., PPC. Although both descending motor and ascending sensory feedback tracts can contribute to the corticomuscular coupling (Meng et al., 2008; Witham et al., 2011), stronger linear coherence was detected at the S1-M1 and motor association cortices (PFA, SMA) compared to the sensory associated PPC. This indicates that linear coupling is mostly related to motor tracts.

This is supported with several previous findings in human and non-human primates. First of all, it was demonstrated that linear information flow in the descending pathways from the S1-M1 and association areas to the periphery is much stronger than that in the ascending pathways (Meng et al., 2008; Tsujimoto et al., 2009; Witham et al., 2010). Secondly, the number of corticospinal tracts originating from the M1 is larger than from other areas, such as SMA and PMA (Dum and Strick, 2002; Maier et al., 2002). Therefore, higher excitatory effect on motoneurons can be exerted, resulting in a stronger linear coupling between S1-M1 and muscles. Although linear coupling between SMA/PMA and muscles are smaller, this is still functionally significant. The linear corticomuscular coupling measured at SMA and PFA was suggested to be functionally related to the fine modulation of force control (Chen et al., 2013).

### Nonlinear Corticomuscular Coupling

Nonlinear coupling between the cortex and the periphery was mainly reported in studies involving sensory input. Harmonic coupling with integer multiples of the stimulation frequencies has been widely reported in the both tactile and proprioceptive studies (Snyder, 1992; Tobimatsu et al., 1999; Jamali and Ross, 2013; Ross et al., 2013; Yang et al., 2016a,b). Recently, Langdon et al. (2011) reported non-integer coupling with the ratio 2:3 of brain response to fingertip stimulation. Our study revealed, for the first time, nonlinear neural coupling between cortical oscillation and EMG during a voluntary motor control task without involving any external sensory input. Similar to linear coupling, the nonlinear corticomuscular coupling was detected in both S1-M1 and sensorimotor association areas. The nonlinear coupling was shown in the similar ratios as previously reported in the somatosensory studies with both integer multiples and the 2:3 non-integer multiple. Since there is no significant “asymmetry” effect, we suggest that the ratios of n:m and m:n likely come from the same type of nonlinearity but appear in a reciprocal to each other due to the closed-loop effect of sensorimotor system (Schouten and Campfens, 2012; Campfens et al., 2014).

Nonlinear neural coupling is thought be associated with synaptic coupling between interneurons (Hyafil et al., 2015). Sensory feedback information is encoded by mechanoreceptors (i.e., muscle spindles and Golgi tendon organs), transmitted through synapses in the dorsal column nuclei, and finally reaches the somatosensory cortex via the thalamo-cortical somatosensory radiation. Compared to corticospinal tracts, the sensory feedback pathways involve more synapses and interneurons. Computational studies based on neural mass and neural field models have demonstrated the nonlinear dynamics of thalamo-cortical system in the sensory pathway, showing similar nonlinearity as we detected in this study (Spiegler et al., 2011; Roberts and Robinson, 2012; Herrmann et al., 2016). Furthermore, the mechanoreceptors such as muscle spindles are also known to be highly nonlinear (Mileusnic et al., 2006). Thus, we suggest that the nonlinear coupling mainly originates from sensory feedback pathway and is mediated in a closed loop.

The processing of sensory information in the cerebral cortex starts from the primary sensory cortices. The information flow does not only pass the sensory associated PPC, but also projects to the primary motor cortex and motor association areas (SMA and PFA; Brovelli et al., 2004; Tsujimoto et al., 2009). As a result, the nonlinear coupling was detected in both S1-M1 and association areas. Different from linear coupling, there are no significant differences of nonlinear coupling shown among different cortical areas. A plausible explanation is that the sensory feedback is essential for closed-loop motor control. Nonlinear coupling between the periphery and the somatosensory cortices may be propagated to the motor-related areas without distortion. In turn, the motor-related areas show similar nonlinear coupling to the muscles.

### EMG Rectification

The surface EMG signal is thought to be a crude representation of many motor unit action potentials from a muscle. EMG rectification has been widely used as a pre-processing step prior to calculating (linear) corticomuscular coherence. Early studies hypothesized this pre-processing could enhance the information of action potential timing and therefore make beta-band corticomuscular coherence more visible (Halliday et al., 1995; Mima and Hallett, 1999). This hypothesis was first validated by Myers et al. (2003) in a simulation study without empirical data. Yao et al. (2007) investigated the effect of EMG rectification on power and coherence spectra using EEG and MEG signals. Their results suggested that EMG rectification possibly improved the identification of motor unit firing rate; however, there was no significant difference between using rectified and non-rectified EMG for corticomuscular coherence estimation. The similar results were also provided by Bayraktaroglu et al. (2011). In agreement with these previous findings, we find no significant difference in linear corticomuscular coupling estimation between using rectified and non-rectified EMG. Notably, there is also no such difference in nonlinear corticomuscular coupling estimation. Our results together with previous findings indicate that EMG rectification is an acceptable pre-processing procedure in corticomuscular coupling estimation but not always necessary, in particular for a static force task.

## Conclusion

This study revealed for the first time nonlinear corticomuscular coupling in the sensorimotor network involving the primary sensorimotor areas and association areas. Our results indicate that corticospinal tracts mainly mediate linear corticomuscular coupling, while nonlinear coupling likely relates to sensory feedback pathways. This work improves our understanding of the neuronal dynamics within sensorimotor system during a voluntary motor task. Additionally, the comparison between using rectified and non-rectified EMG provides first empirical evidence indicating that EMG rectification is an acceptable but unnecessary pre-processing step for computing nonlinear corticomuscular coupling during a static force task. Our results on nonlinear corticomuscular coupling could provide a reference for future clinical studies, and the nonlinear analysis approach could serve as a general tool to characterize the nonlinear coupling in neural systems.

## Author Contributions

YY conducted the whole study and drafted the manuscript. YY and ACS contributed in problem identification. YY and TS-E collected the experimental data. YY, TS-E, MR, FCTH and ACS participated in editing the manuscript.

## Conflict of Interest Statement

The authors declare that the research was conducted in the absence of any commercial or financial relationships that could be construed as a potential conflict of interest.

## Footnotes

^1^http://www.talairach.org/
